# Predictive Significance of the Prognostic Nutritional Index (PNI) in Patients with Severe COVID-19

**DOI:** 10.1155/2021/9917302

**Published:** 2021-07-09

**Authors:** Wei Wei, Xingyue Wu, Chaoyuan Jin, Tong Mu, Guorong Gu, Min Min, Sucheng Mu, Yi Han

**Affiliations:** ^1^Department of Emergency Medicine, Zhongshan Hospital, Fudan University, Shanghai, China; ^2^Shanghai Medical College, Fudan University, Shanghai, China; ^3^Nanjing Sport Institute, Nanjing, China

## Abstract

**Background:**

The prognostic nutritional index (PNI) has been reported to significantly correlate with poor survival and postoperative complications in patients with various diseases, but its relationship with mortality in COVID-19 patients has not been addressed.

**Method:**

A multicenter retrospective study involving patients with severe COVID-19 was conducted to investigate whether malnutrition and other clinical characteristics could be used to stratify the patients based on risk.

**Results:**

A total of 395 patients were included in our study, with 236 patients in the training cohort, 59 patients in the internal validation cohort, and 100 patients in the external validation cohort. During hospitalization, 63/236 (26.69%) and 14/59 (23.73%) patients died in the training and validation cohorts, respectively. PNI had the strongest relationships with the neutrophil-lymphocyte ratio (NLR) and lactate dehydrogenase (LDH) level but was less strongly correlated with the CURB65, APACHE II, and SOFA scores. The baseline PNI score, platelet (PLT) count, LDH level, and PaO_2_/FiO_2_ (P/F) ratio were independent predictors of mortality in COVID-19 patients. A nomogram incorporating these four predictors showed good calibration and discrimination in the derivation and validation cohorts. A PNI score less than 33.405 was associated with a higher risk of mortality in severe COVID-19 patients in the Cox regression analysis.

**Conclusion:**

These findings have implications for predicting the risk of mortality in COVID-19 patients at the time of admission and provide the first direct evidence that a lower PNI is related to a worse prognosis in severe COVID-19 patients.

## 1. Introduction

By the end of 19^th^ June 2021, more than 177,833,450 confirmed coronavirus disease 2019 (COVID-19) cases had been documented worldwide, with more than 3,851,736 deaths [1]. Unlike patients with other common infectious diseases, patients with COVID-19 have a wide range of clinical manifestations, including complex and mixed pulmonary conditions and multiorgan failure that can lead to death. However, not all patients develop a poor clinical outcome. Given the large number of COVID-19 cases, we need to pay more attention to those who are likely to progress to death.

Recent evidence has shown that malnutrition is a critical prognostic factor in many diseases, including autoimmune diseases [2], cardiovascular diseases [3], lung diseases [4, 5], and malignancies [6]. Chronic inflammatory diseases are associated with the increased production of catabolic cytokines, muscle catabolism, appetite suppression, and lower albumin levels [7]. High degrees of malnutrition correlate with high levels of inflammation [8]. Malnutrition is a modifiable risk factor [9].

The prognostic nutritional index (PNI) is calculated based on the serum albumin concentration and lymphocyte count in the peripheral blood. Previously, the PNI was reported to correlate significantly with poor survival and postoperative complications in patients with various malignant digestive system tumors [10, 11]. A previous study indicated that a lower PNI in patients with a decreased left atrial ejection fraction tended to be associated with a higher risk of mortality in a retrospective study [12]. Once they have been infected with severe acute respiratory syndrome coronavirus 2 (SARS-CoV-2), patients, particularly elderly patients, often develop cardiovascular dysfunction due to the widespread expression of angiotensin-converting enzyme 2 (ACE2) in the heart and blood vessels [13]. Therefore, the prognostic value of the PNI in patients with COVID-19 is worth further investigation. However, to date, the relevant studies have mainly focused on crude analyses rather than establishing a systematic, quantified model; hence, the application value of the present PNI is extremely limited [14, 15].

As current evidence regarding the prognostic impact of malnutrition on severe COVID-19 is limited and the relationship between malnutrition and mortality in COVID-19 patients has not been addressed, we aimed to identify the prevalence, clinical associations, and prognostic consequences of malnutrition in a retrospective cohort of patients with severe COVID-19 and establish a novel prognostic nomogram for the early prediction and modification of the disease outcome.

## 2. Materials and Methods

### 2.1. Data Source

The medical records and compiled data used in this retrospective study were collected from COVID-19 patients in Renmin Hospital of Wuhan University and Jin Yin Tan Hospital in Wuhan City. All patients had a clear clinical outcome of either hospital discharge or death. Data were reviewed by a trained team of physicians [16]. The study was approved by the Ethics Committee of Renmin Hospital of Wuhan University.

### 2.2. Laboratory Confirmation

Laboratory confirmation of SARS-CoV-2 infection was obtained with patients' throat swab specimens and was conducted in Renmin Hospital of Wuhan University and Jin Yin Tan Hospital. The severity of COVID-19 was defined at the time of admission, according to American Thoracic Society guidelines for community-acquired pneumonia (CAP) [17]. All laboratory tests were performed according to the clinical care needs of the patients. The laboratory assessments consisted of a complete blood count, liver function assessment, and arterial blood gas measurements. To minimize sampling bias, the data on admission were obtained, by communicating effectively with medical workers and double-checking.

The neutrophil-lymphocyte ratio (NLR) was defined by dividing the neutrophil count by the lymphocyte count. The prognostic nutritional index (PNI) score was calculated using the formula ten × serum albumin (g/dL) + 0.005 × total lymphocyte count (mm^3^) [18]. A score greater than 38 is considered normal; scores of 35 to 38 and less than 35 reflect moderate and severe malnutrition, respectively.

### 2.3. Statistical Analysis

Continuous variables are expressed as the medians with interquartile ranges (IQRs) as well as mean with standard deviation, while categorical variables are presented as frequencies and percentages (%). To determine differences between the two groups, chi-squared tests were performed for categorical variables, and Wilcoxon rank-sum and one-way ANOVA tests were performed for continuous variables [19].

A novel prognostic nomogram was constructed based on the results of multivariate analysis obtained with the rms package in R; the nomogram was developed based on 80% of the internal data and validated with the remaining 20% of internal data and another 100 external cases. The discrimination performance of the nomogram was quantified using the concordance index (*C*-index) and calibration curve analysis. The *C*-index value ranges from 0.5 to 1.0, with 0.5 indicating random chance and 1.0 demonstrating perfect discrimination.

To evaluate the discriminatory ability of the prognostic nomogram, receiver operating characteristic (ROC) curves were generated, and differences among the areas under the curve (AUCs) were compared. Correlations were assessed with Kendall's tau-b analysis, and survival probability was evaluated by Cox analysis.

All analyses were conducted using R (version 3.6.3) and SPSS (version 25). *P* values less than 0.05 were considered statistically significant in each statistical analysis.

## 3. Results

### 3.1. Clinical Characteristics of Patients with COVID-19

**Figure 1:**
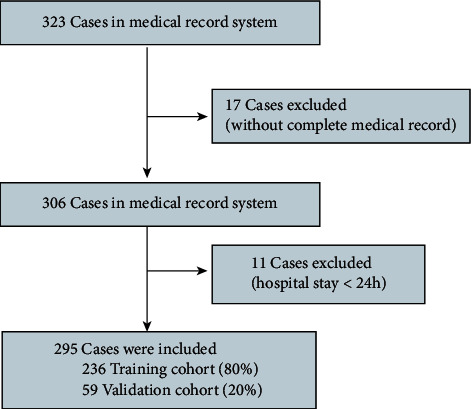
Flowchart of patient recruitment in the internal cohort.

A total of 323 patients with severe COVID-19 were identified according to the inclusion criteria, of whom 28 patients were excluded for having (1) incomplete medical records (*n* = 17) or (2) hospital stays less than 24 h (*n* = 11). Finally, 295 patients were included in our study, with 236 patients in the training cohort and 59 patients in the validation cohort (Figure 1). The clinical characteristics of the patients included in the training and validation cohorts are presented in Table 1. Among them, 116 (49.15%) and 37 (62.71%) patients were male, with a median age of 61 years and 60 years in the training and validation cohorts, respectively. There were no significant differences in clinical characteristics or disease severity scores (using the Acute Physiology and Chronic Health Evaluation II (APACHE II), sequential organ failure assessment (SOFA), and CURB65 scores) between the two cohorts (Table 1). During hospitalization, 63/236 (26.69%) and 14/59 (23.73%) patients died in the training and validation cohorts, respectively. In addition, 218 (73.90%) patients recovered and had been discharged at the time of analysis.

### 3.2. Comparison between Surviving and Nonsurviving Patients in the Training Cohort

Surviving and nonsurviving patients with COVID-19 had significant differences in many clinical characteristics and laboratory indicators on admission. Patients in the nonsurviving group were significantly older (74 years, IQR 63-81) than surviving patients (55 years, IOR 44-66). Although many studies have indicated that male patients with COVID-19 had a higher risk of mortality, in the current study, 37 of the 63 (58.73%) patients in the nonsurviving group were male, which was not different from the proportion in the surviving group (79/173, 45.66%, *P* > 0.05). Patients with hypertension were more likely to die, and those with dyspnea before admission tend to have a poor outcome (*P* < 0.001), with a history of hypertension in 41/63 (65.08%) of nonsurviving patients and 63/171 (36.84%) of surviving patients and dyspnea in 31/63 (49.21%) of nonsurviving patients and 36/171 (21.05%) of surviving patients. No differences were observed in headache, cough, and diarrhea.

In addition, the white blood cell count (10^9^/L) was 8.49 (IQR 5.61-11.98) in nonsurviving patients, which was dramatically higher than that in surviving patients (5.65 (IQR 4.28-8.10)). Meanwhile, the lymphocyte count (10^9^/L) was 0.60 (IQR 0.41-1.13) in nonsurviving patients, which was dramatically lower than that in surviving patients (1.53 (IQR 0.90-11.2)). The monocyte count (10^9^/L) was 0.42 (IQR 0.28-0.65) in nonsurviving patients, which was dramatically lower than that in surviving patients (0.57 (IQR 0.39-4.8)) (Table 2, *P* < 0.001). As an indicator of the response to an infection, the neutrophil/lymphocyte ratio (NLR) was also higher in nonsurviving patients (12.51, IQR 7.22-18.83) than in surviving patients (3.41, IQR 1.89-7.16). The blood platelet count represents coagulation function and was dramatically reduced in nonsurviving patients (155.00 (IQR 114.50-208.50) vs. 35.90 (IRQ 157.00-287.00)) (*P* < 0.001). Moreover, the serum levels of AST and GLU were also higher in patients with poor outcomes than in the other group (41.00 (IRQ 24.00-63.00) vs. 30.00 (IRQ 22.00-42.00), *P* < 0.001; 7.00 (IRQ 5.80-10.30) vs. 5.60 (IRQ 4.96-7.13), *P* < 0.001). COVID-19 damages respiratory function, and the PaO_2_/FiO_2_ (P/F) ratio in nonsurviving patients was markedly lower than that in surviving patients (104.44 (IQR 75.56-150.0) vs. 257.58 (IQR 168.85-314.75)). The PNI score in nonsurviving patients on admission was 33.41 (IQR 31.46-36.63), which was significantly lower than that in surviving patients (37.21 (IQR 34.07-40.56)) (Table 2).

### 3.3. Logistic Regression Analysis and Nomogram Establishment

Multivariate logistic regression analysis showed that the PLT count (odds ratio (OR) and 95% confidence interval (CI), 0.984 (0.974, 0.993); *P* = 0.001), baseline PNI score (OR and 95% CI, 0.853 (0.740, 0.983); *P* = 0.028), lactate dehydrogenase (LDH) level (OR with 95% CI, 1.005 (1.001, 1.009); *P* = 0.012), and P/F ratio (OR with 95% CI, 0.988 (0.981, 0.995); *P* = 0.001) were independent predictors of mortality in COVID-19 patients (Table 3).

**Figure 2:**
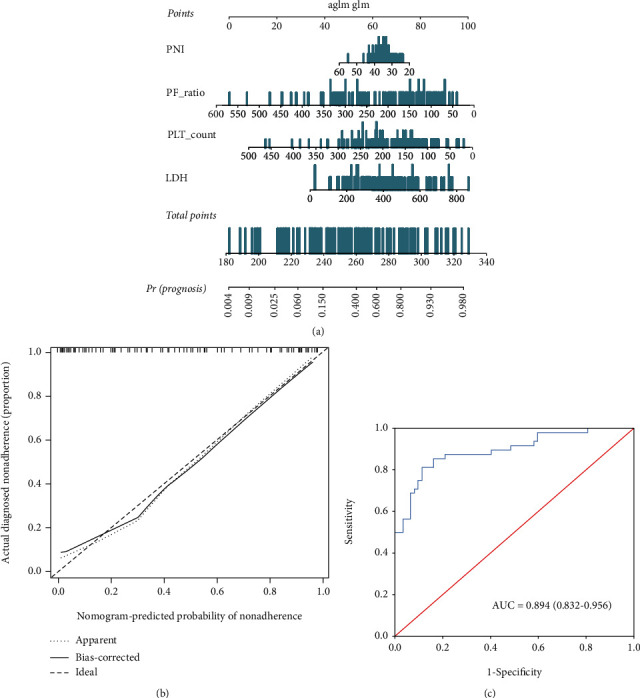
Development and performance of the nomogram in the training cohort. (a) A nomogram for predicting the prognosis of patients with severe COVID-19; (b) calibration curve of the nomogram in the training cohort, which depicts the calibration of the nomogram in terms of the agreement between the predicted risk of death and observed outcomes. The 45° dotted line represents an ideal prediction, and the solid line represents the bias-corrected predictive performance of the nomogram. The closer the solid line fits the ideal line, the better the predictive accuracy of the nomogram; (c) ROC curve of the nomogram in the training cohort.

**Figure 3:**
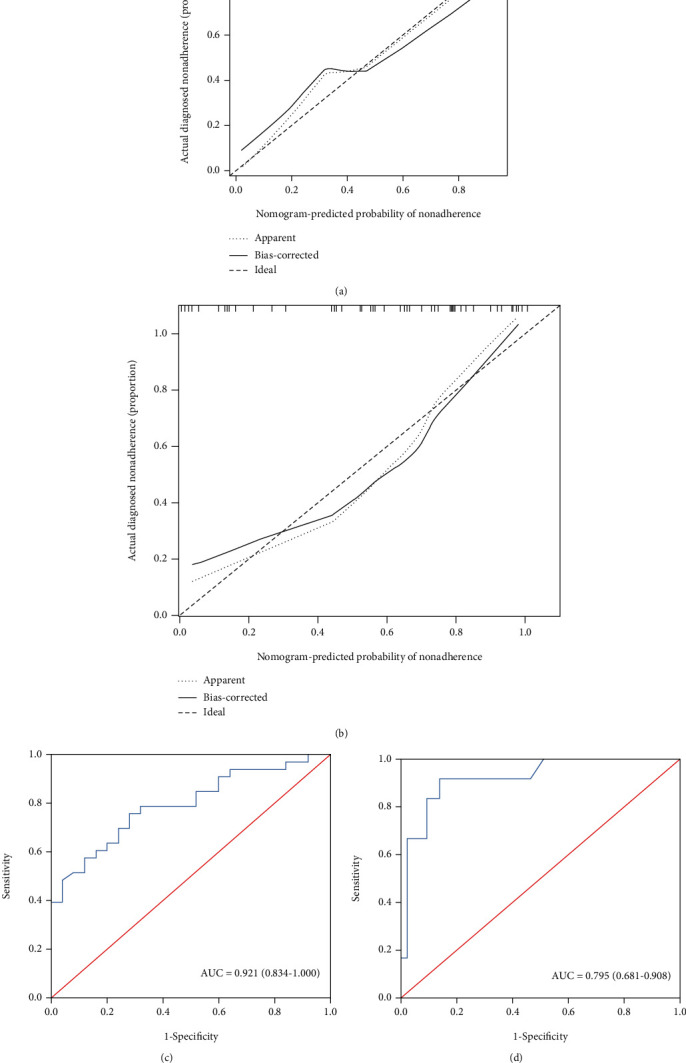
Performance of the nomogram in the validation cohorts. Calibration curve of the nomogram in the internal (a) and external (b) validation cohorts. The 45° dotted line represents an ideal prediction, and the solid line represents the bias-corrected predictive performance of the nomogram. The closer the solid line fits the ideal line, the better the predictive accuracy of the nomogram; ROC curves of the nomogram in the internal (c) and external (d) validation cohorts, respectively.

A nomogram incorporating these four predictors was then constructed (Figure 2(a)) and showed good reliability (*C*-index: 0.959). The calibration curve for the nomogram (Figure 2(b)) showed good calibration in the training cohort. Then, the favorable calibration of the nomogram was confirmed in the validation cohort (Figure 3(a)). The AUCs of the nomogram in the training and validation cohorts were 0.894 (95% CI, 0.832-0.956; Figure 2(c)) and 0.921 (95% CI, 0.834-1.000; Figure 3(c)), respectively, which revealed good discrimination. Furthermore, our nomogram also performed well in an external validation cohort (Figure 3(b)), with an AUC of 0.795 (95% CI, 0.681-0.908, Figure 3(d)).

### 3.4. Correlation between the PNI Score and Other Biomarkers of Disease Severity

**Figure 4:**
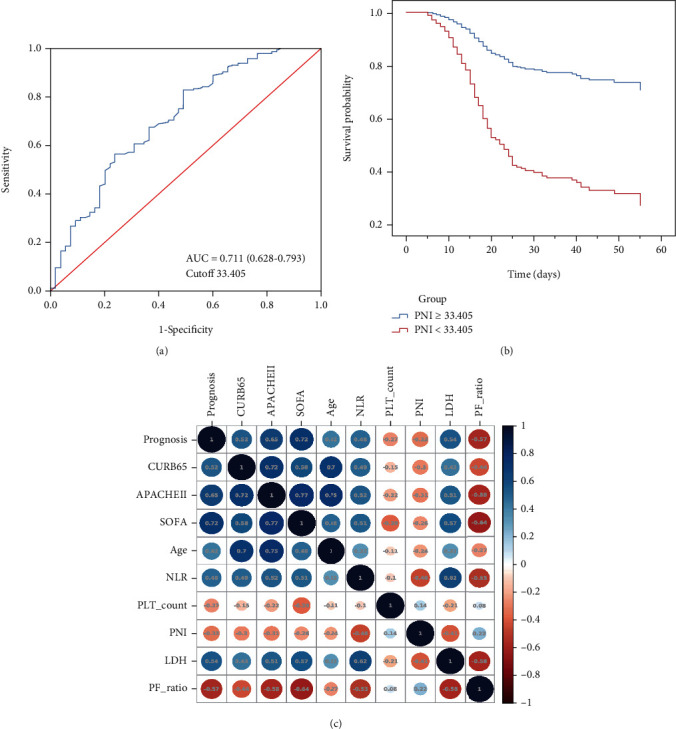
The significance of the PNI score for the prediction of mortality in patients with severe COVID-19. (a) ROC curve of PNI; (b) Cox analysis of COVID-19 patients based on the cutoff value of the PNI score; (c) correlation between the PNI score and other indicators of disease severity.

The AUC for the PNI score was 0.711 (95% CI, 0.628-0.793), with a cutoff value of 33.405 (Figure 4(a)). We then divided the enrolled data into two groups based on the cutoff value of the PNI score. Cox analysis showed a significant reduction in the survival probability in the patients with a PNI score less than 33.405 on admission (Figure 4(b)). Furthermore, the correlation between the PNI score and disease severity was evaluated with Kendall's tau-b analysis. The results showed that the PNI score had the strongest negative relationships with the NLR and LDH level (*R* = −0.458, *P* < 0.001 and *R* = −0.414, *P* < 0.001) but was less strongly correlated with the CURB65 (*R* = −0.303, *P* < 0.001), APACHE II (*R* = −0.313, *P* < 0.001), and SOFA (*R* = −0.256, *P* < 0.001) scores (Figure 4(c)).

## 4. Discussion

In this study, we retrospectively assessed the clinical characteristics of severe COVID-19 patients from multiple hospitals and identified the baseline risk factors for mortality. Our results indicated that the PLT count, PNI score, P/F ratio, and LDH level on admission were independent predictors of mortality. The nomogram based on these risk factors showed good calibration and discrimination in the training and validation cohorts.

With the rapid increase in newly confirmed and severe cases, the management of patients with severe cases has become a challenging issue during the COVID-19 outbreak. The timely identification of patients at a high risk of developing acute respiratory distress syndrome (ARDS), multiorgan failure, and death might help clinicians develop individual treatment plans and rationally allocate medical resources.

In our cohorts, the mortality of patients with severe COVID-19 was 26.70%, which was higher than that in some large-scale reports [20] and slightly lower than that in Washington state in February [21]. This might be because we enrolled patients with COVID-19 during the early phase of the pandemic in Wuhan City, China. In addition, we found that nonsurviving patients were more likely to be older and have underlying hypertension than surviving patients, which is in agreement with recent reports, which have suggested that age and hypertension may be risk factors for progression to severe COVID-19 [22, 23].

In our study, the patients with severe COVID-19 who died had lower baseline platelet counts, PNI scores, and P/F ratios and higher LDH levels, and these variables were independent risk factors for mortality. Previous studies showed that thrombocytopenia in COVID-19 patients was not a significant predictor of disease progression or adverse outcomes [24, 25]. Studies in consecutive patients with COVID-19 have reported that only approximately 5% of patients present with a platelet count less than 100 × 10^9^ cells/L. Mild thrombocytopenia (a platelet count < 150 × 10^9^ cells/L) is identified in 70-95% of patients with severe COVID-19 [20]. The P/F ratio, which directly reflects lung oxygenation, represents the severity of ARDS in COVID-19 patients. In our study, the P/F ratio in nonsurviving patients was twofold lower than that in surviving patients, which was similar to the findings in Arentz et al.'s study [21]. Furthermore, in our previous study, the area under the curve (AUC = 0.878) implied that a serum LDH level greater than 344.5 U/L was strongly predictive of severe COVID-19, with high specificity (96.9%) and sensitivity (68.8%), further confirming that the LDH level is a strong predictive factor that can be used for the early detection of lung injury and severe COVID-19 cases [26].

Interestingly, we found that the prognostic nutritional index (PNI), which was initially used to assess patients' immune and nutritional statuses during the perioperative period and is calculated based on the serum albumin concentration and lymphocyte count in the peripheral blood, was also associated with mortality in patients with severe COVID-19. Previously, PNI was reported to be significantly correlated with poor survival and postoperative complications in patients with various malignant digestive system tumors [10, 11]. However, no studies have explored the association between the immunonutritional status and prognosis in COVID-19 patients.

Albumin is a widely used indicator of nutrition and has been shown to be associated with a poor outcome in critically ill patients. Growing evidence has shown that COVID-19 is associated with a strong cytokine storm [27] and, consequently, the consumption of albumin. Hypoalbuminemia is a typical clinical manifestation of various critical illnesses [28–31]. In our study, we found that the PNI score was significantly lower in nonsurviving patients and was most strongly negatively related to the NLR. The NLR is a reliable marker of systemic inflammation. A higher NLR has been widely reported to be a predictive indicator of poor survival in patients with many different diseases [32, 33]. The PNI score involves a combination of the albumin level and lymphocyte count in the peripheral blood, whereas the NLR can only reflect the inflammation status. In recent studies, the PNI was superior to the NLR as a prognostic marker in many cancer patients [34–36].

Furthermore, we also showed that the PNI score was negatively correlated with the LDH level. The LDH level was found to be positively associated with the C-reactive protein (CRP) level and negatively associated with the lymphocyte count [26]. Therefore, the PNI has been confirmed to be a marker of the immunonutritional status of critically ill patients. The PNI was less strongly correlated with the CURB65, APACHE II, and SOFA scores in our study. The PNI score is a new biomarker of critical illness.

A combination of nutrition and inflammation can better predict the disease progression than an individual predictor. However, studies on the PNI score in COVID-19 patients are extremely limited. We evaluated the clinical characteristics and prognostic importance of the PNI score in severe COVID-19 patients, providing the first direct evidence that a lower PNI score is related to a worse prognosis. Furthermore, our nomogram, which includes the PNI score, had a higher AUC than the PNI score alone for predicting disease prognosis, which provides a new method of evaluating disease outcomes with good predictive accuracy.

However, there are still some limitations of our study. Our study was a retrospective study. The characteristics of the enrolled patients were imbalanced, and approximately 26.70% of the patients died. Second, the sample size was limited, and adjuvant treatments during hospitalization were thought to be similar but were not analyzed. A larger global cohort study of patients with COVID-19 would help further validate the nomogram model and identify the risk factors for severe COVID-19 and mortality.

## 5. Conclusion

In conclusion, our results provide the first direct evidence that a lower PNI score is related to a worse prognosis in patients with severe COVID-19. We also found that a lower PLT count, PNI, and P/F ratio and a higher LDH level on admission are independent predictors of mortality in patients with severe COVID-19, and the nomogram based on these four risk factors showed good predictive accuracy in the training and validation cohorts.

## Figures and Tables

**Table 1:** Clinical characteristics of patients with COVID-19 in the training and validation cohorts.

	Training cohort (*N* = 236)	Validation cohort (*N* = 59)	*P* value
Gender (*N*, %)			0.062
Male	116 (49.15%)	37 (62.71%)	
Female	120 (50.85%)	22 (37.29%)	
Age (years)	61 (48,71)	60 (47.69)	0.550
History (*N*, %)			
Hypertension	70 (29.67%)	15 (25.42%)	0.520
DM	36 (15.25%)	8 (13.56%)	0.744
Symptoms (*N*, %)			
Fatigue	104 (44.07%)	27 (45.76%)	0.815
Headache	15 (6.36%)	3 (5.08%)	0.952
Cough	152 (64.41%)	42 (71.19%)	0.326
Dyspnea	67 (28.39%)	19 (32.20%)	0.564
Diarrhea	27 (11.44%)	6 (10.17%)	0.774
Signs			
Temperature	36.70 (36.50, 37.00)	36.60 (36.40, 37.00)	0.548
HR (bpm)	86 (80, 98)	88 (81, 98)	0.336
RR (/min)	20 (19, 24)	20 (20, 25)	0.552
Laboratory indices			
WBC counts (10^9^/L)	6.29 (4.46, 9.37)	5.37 (3.96, 8.02)	0.105
NEU counts (10^9^/L)	6.51 (3.56, 50.3)	5.14 (3.12, 29.41)	0.244
LYM counts (10^9^/L)	1.21 (0.72, 5.33)	1.16 (0.72, 2.77)	0.886
MON counts (10^9^/L)	0.53 (0.37, 2.33)	0.53 (0.33, 1.15)	0.764
NLR	4.62 (2.32, 10.02)	3.78 (1.79, 9.11)	0.406
HGB (g/L)	125.00 (115.00, 137.00)	130.00 (119.00, 143.00)	0.088
PLT (10^9^/L)	199.50 (141.80, 262.30)	205.00 (156.00, 252.00)	0.529
ALB (g/L)	35.10 (32.00, 39.40)	37.00 (32.30, 40.00)	0.308
PNI	36.19 (33.23, 39.78)	37.00 (32.30, 40.00)	0.916
ALT (U/L)	26.50 (17.30, 43.00)	27.00 (16.50, 41.50)	0.689
AST (U/L)	31.00 (22.00, 46.00)	30.00 (21.00, 38.50)	0.460
GLU (mmol/L)	5.90 (5.02, 7.79)	5.56 (4.77, 6.79)	0.059
LDH (U/L)	296.50 (211.00, 432.50)	273.00 (220.00, 420.00)	0.825
P/F ratio (mmHg)	168.85 (100.42, 289.75)	231.42 (131.11, 342.00)	0.120
CURB65	1.0 (0, 1.0)	1.0 (0, 1.0)	0.931
APACHE II	6.0 (3.0, 10.0)	6.0 (3.0, 10.0)	0.600
SOFA	2.0 (1.0, 4.0)	1.0 (0, 3.0)	0.143
Death (*N*, %)	63 (26.69%)	14 (23.73%)	0.643

Abbreviations: DM; diabetes mellitus; WBC: white blood cells; NEU: neutrophils; LYM: lymphocytes; MON: monocytes; NLR: neutrophil-lymphocyte ratio; HGB: hemoglobin; PLT: platelet; ALB: albumin; PNI: prognostic nutritional index; ALT: alanine transaminase; AST: aspartate aminotransferase; LDH: lactic dehydrogenase; P/F ratio: PaO_2_/FiO_2_ ratio; CURB65: confusion, uremia, respiratory rate, blood pressure; APACHE II: Acute Physiology and Chronic Health Evaluation II; SOFA: sequential organ failure assessment.

**Table 2:** Clinical characteristics of surviving and nonsurviving patients in the training cohort.

	Surviving patients (*N* = 173)	Nonsurviving patients (*N* = 63)	*P* value
Gender (*N*, %)			0.076
Male	79 (45.66%)	37 (58.73%)	
Female	94 (54.34%)	26 (41.27%)	
Age (years)	55 (44, 66)	74 (63, 81)	<0.001
History (*N*, %)			
Hypertension	40 (23.12%)	30 (47.62%)	<0.001
DM	25 (14.45%)	11 (17.46%)	0.569
Symptoms (*N*, %)			
Fatigue	63 (36.42%)	41 (65.08%)	<0.001
Headache	13 (7.51%)	2 (3.17%)	0.227
Cough	115 (66.47%)	37 (58.73%)	0.272
Dyspnea	36 (20.81%)	31 (49.21%)	<0.001
Diarrhea	20 (11.56%)	7 (11.11%)	0.925
Signs			
T	36.70 (36.50, 37.00)	36.70 (36.40, 36.90)	0.543
HR	86 (80, 98)	86 (77,102)	0.898
RR	20 (19, 22)	20 (19, 28)	0.052
Laboratory indices			
WBC counts (10^9^/L)	5.65 (4.28, 8.10)	8.49 (5.61, 11.98)	<0.001
NEU counts (10^9^/L)	5.51 (3.02, 61.85)	7.89 (4.62, 13.16)	0.259
LYM counts (10^9^/L)	1.53 (0.9, 11.2)	0.60 (0.41, 1.13)	<0.001
MON counts (10^9^/L)	0.57 (0.39, 4.8)	0.42 (0.28, 0.65)	<0.001
NLR	3.41 (1.89, 7.16)	12.51 (7.22, 18.83)	<0.001
HGB (g/L)	125.00 (116.50, 136.00)	122.00 (111.00, 137.50)	0.560
PLT (10^9^/L)	214.00 (157.00, 287.00)	155.00 (114.50, 208.50)	<0.001
ALB	35.90 (32.60, 39.90)	33.40 (31.40, 36.60)	0.004
PNI	37.21 (34.07, 40.56)	33.41 (31.46, 36.63)	<0.001
ALT (U/L)	27.00 (16.50, 43.00)	25.00 (20.00, 45.00)	0.708
AST (U/L)	30.00 (22.00, 42.00)	41.00 (24.00, 63.00)	0.001
GLU (mmol/L)	5.60 (4.96, 7.13)	7.00 (5.78, 10.30)	<0.001
LDH (U/L)	263.00 (195.00, 355.00)	493.00 (362.50, 611.50)	<0.001
P/F ratio	257.58 (168.85, 314.75)	104.44 (75.56, 150.00)	<0.001

Abbreviations: DM: diabetes mellitus; WBC: white blood cells; NEU: neutrophils; LYM: lymphocytes; MON: monocytes; NLR: neutrophil/lymphocyte ratio; HGB: hemoglobin; PLT: platelet; ALB: albumin; PNI: prognostic nutritional index; ALT: alanine transaminase; AST: aspartate aminotransferase; LDH: lactic dehydrogenase; P/F ratio: PaO_2_/FiO_2_ ratio; CURB65: confusion, uremia, respiratory rate, blood pressure; APACHE II: Acute Physiology and Chronic Health Evaluation II; SOFA: sequential organ failure assessment.

**Table 3:** Univariate and multivariate logistic analysis of potential prognostic factors.

Factors	Univariate analysis		Multivariate analysis	
	OR (95% CI)	*P* value	OR (95% CI)	*P* value
Age	1.089 (1.060, 1.119)	<0.001		0.105
Hypertension	3.023 (1.646, 5.551)	<0.001		0.165
Fatigue	3.254 (1.780, 5.950)	<0.001		0.666
Dyspnea	3.687 (1.992, 6.822)	<0.001		0.373
WBC count	1.197 (1.101, 1.301)	<0.001		0.696
NLR	1.155 (1.101, 1.212)	<0.001		0.805
PLT count	0.991 (0.987, 0.995)	<0.001	0.984 (0.974, 0.993)	0.001
PNI	0.839 (0.774, 0.911)	<0.001	0.853 (0.740, 0.983)	0.028
LDH	1.009 (1.006, 1.011)	<0.001	1.005 (1.001, 1.009)	0.012
P/F ratio	0.986 (0.981, 0.992)	<0.001	0.988 (0.981, 0.995)	0.001

## Data Availability

You could find these data in our results.

## Abbreviations

PNI: Prognostic nutritional index
COVID-19: Coronavirus disease 2019
SARS-CoV-2: Severe acute respiratory syndrome coronavirus 2
CAP: Community-acquired pneumonia
NLR: Neutrophil-lymphocyte ratio
IQR: Interquartile range
C-index: Concordance index
ROCs: Receiver operating characteristics
AUC: Areas under the curve
APACHE II: Acute Physiology and Chronic Health Evaluation II
SOFA: Sequential organ failure assessment
WBC: White blood cell
AST: Aspartate aminotransferase
LDH: Lactic dehydrogenase
P/F: PaO_2_/FiO_2_
PLT: Lower platelet
OR: Odds ratio
ARDS: Acute respiratory distress syndrome
CI: Confidence interval
CRP: C-reactive protein.
